# Effects of an advanced first aid course or real-time video communication with ambulance personnel on layperson first response for building-site severe injury events: a simulation study

**DOI:** 10.1186/s12873-023-00917-4

**Published:** 2024-01-07

**Authors:** Hans Hedberg, Pia Hedberg, Jonas Aléx, Sofia Karlsson, Michael Haney

**Affiliations:** 1 Anesthesiology and Intensive Care Medicine, Surgical and Perioperative Sciences, Umeå University, Umeå, Sweden; 2 Nursing and Surgical and Perioperative Sciences, Center for Disaster Medicine, Umeå University, Umeå, Sweden; 3 Nursing, Umeå University, Umeå, Sweden; 4 Surgical and Perioperative Sciences, Surgery, Umeå University, Umeå, Sweden

**Keywords:** Construction Safety, Workplace incident, Prehospital trauma, First aid training, Layperson, Bystander, Telemedicine, Video

## Abstract

**Background:**

The risk of high-energy trauma injuries on construction sites is relatively high. A delayed response time could affect outcomes after severe injury. This study assessed if an advanced first aid course for first aid response for laypersons (employees or apprentices) in the construction industry or real-time video communication and support with ambulance personnel, or neither, together with access to an advanced medical kit, would have an effect on immediate layperson vital responses in a severe injury scenario.

**Method:**

This was a controlled simulation study. Employees or apprentices at a construction site were recruited and randomly allocated into a group with video support or not, and advanced first aid course or not, and where one group had both. The primary outcomes were correct behavior to recognize and manage an occluded airway and correct behavior to stop life-threatening bleeding from a lower extremity injury. Secondary outcomes included head-to-toe assessment performed, placement of a pelvic sling, and application of remote vital signs monitors.

**Results:**

Ninety participants were included in 10 groups of 3 for each of 4 exposures. One group was tested first as a baseline group, and then later after having done the training course. Live video support was effective in controlling bleeding. A first aid course given beforehand did not seem to be as effective on controlling bleeding. Video support and the first aid course previously given improved the ability of bystanders to manage the airway, the combination of the two being no better than each of the interventions taken in isolation. Course exposure and video support together were not superior to the course by itself or video by itself, except regarding placing the biosensors on the injured after video support. Secondary results showed an association between video support and completing a head-to-toe assessment. Both interventions were associated with applying a pelvic sling.

**Conclusion:**

These findings show that laypersons, here construction industry employees, can be supported to achieve good performance as first responders in a major injury scenario. Prior training, but especially live video support without prior training, improves layperson performance in this setting.

**Supplementary Information:**

The online version contains supplementary material available at 10.1186/s12873-023-00917-4.

## Background

Serious high-energy injury events are a risk to construction sites workers, and a higher risk in that compared to other industries [1–2]. This is partly due to the varying work environment and sometimes rapidly changing construction activities. The work environment on construction sites can be stressful for workers [3]. In Sweden, approximately 1000 serious injury events are reported per year, and each year some resulting in fatalities [4].

In the event of severe injury events on construction sites where there is one injured person, there are several immediate aspects needed for good response, including trying to stop catastrophic bleeding if possible and establishing a patent airway if needed. Even before this at an injury event, evaluation and awareness of the situation and mechanism of injury is done, meaning that there is a determination that the site is safe enough to stay deliver peer rescue and prevent injuries to responders [5]. Definitive medical measures then need to be implemented as quickly as possible [6–7]. The first life-saving measures are the same in all healthcare systems and settings [5, 8, 9].

In Sweden, the response time for ambulances has increased by more than two minutes per year over the past 10 years. In 2021, the response time for ambulances was 18 min on average for all alarms [10], though this largely reflects responses in major population centers. A delayed response time will affect the trauma care response and outcomes after severe trauma injury if there is no other care [11]. Laypersons can provide lifesaving procedures before the ambulance arrives. Bakke et al., 2015 observed 330 prehospital trauma alarms and responses, and noted that in that cohort 35% of the first-aid providers had participated in a first aid training course. In that report, bystanders with documented first aid training course gave better first aid than those with unknown competence in first aid [12]. Given a long ambulance response time to remote location or where ambulance resources are scarce, live video communication with ambulance personnel may hold promise for supporting layperson at the injury event. Further, if continuous vital sign assessment through biosensors which laypersons could be applied, and then signals received by ambulance personnel, these could inform video support for guidance of laypersons on site [13].

Initial assessments and responses from laypersons have been shown to be of benefit especially in cardiac arrest situation and cardiopulmonary resuscitation, but also in prehospital trauma care [14–16]. Remote consultation for fire brigade or police responders with paramedics or emergency doctors has been described [17–20]. It is not known if remote video support for laypersons in advanced first aid and trauma resuscitation, for example in the event of a remote workplace injury event can be beneficial. Also, for laypersons in remote settings where advanced first aid might be needed, it is not well understood how well an advanced first aid course and access to usual health care system resuscitation equipment might help to facilitate better layperson first responses to a severe injury event.

The general study question was if, for laypersons, a first aid training course or a direct video support system could have benefit on first aid trauma responses during the first 10 min, with focus on catastrophic bleeding and airway management. In a setting where an advanced medical kit is available for first responders, the primary hypothesis was that access to direct video support from remote ambulance personnel would be associated with a higher level of performance in the two immediate layperson critical behaviors, which are correct recognition and behavioral responses to catastrophic bleeding and occluded airway, compared to first responders who did not have remote video support from ambulance personnel. A second hypothesis that the combination of a layperson training course, together with video support from ambulance personnel, would be associated with superior performance with the two critical behaviors compared to either the training course by itself or video support by itself. A third hypothesis was that video support by itself during the layperson first response would be superior to previous training course experience by itself concerning the early critical behaviors. Our objective was to test these hypotheses with actual construction workers or apprentices on site with a simulated major injury scenario using a full-scale human patient simulator, and where groups would have either no beforehand advanced first aid course, or a course by itself, and for direct video support by itself or a combination of the two.

## Methods

### Study design

This was a controlled simulation study. With cooperation from building companies or training program and sites in northern Sweden, persons consenting to participate in the study were scheduled for test days in groups of 3 persons at a time at their building site. This was presented as a training course and a part of this study. All the groups had the same first day activity with a scenario-based practical assessment without training and then the planned course. After that, half of the groups were recalled for a second assessment after 4–6 weeks, where they were allocated to the treatment groups based on when they scheduled their second assessment. Those that scheduled for the first 10 assessment days were allocated to treatment group 3 and those that were scheduled for the last 10 assessment days were included in treatment group 4. The treatment or exposures were not known to the companies or participants ahead of time. Prospective randomization by lot was not chosen due to challenges for the building companies in scheduling the days, and this minimization method was used to achieve balance for groups and exposures, even if this was not formal block randomization.

Each ‘treatment’ group (of 3 individuals) was planned to comprise one of 10 teams for each ‘treatment’ arm. The treatments or exposures were as follows: Group 1 no course training and no video support during the simulation-based assessment. This same group then later (after the assessment) went further and participated in the training course and had a second assessment 4 to 6 weeks after their course, and this assessment group was called Group 4. Group 2 and Group 3 had video support during the assessment, Group 2 without pre-treatment training course and Group 3 with the completed training course prior to their assessment. All groups had access to standardized medical equipment during the simulation-based assessment, but which was a kit with which no participants had familiarity before the study. All groups participated in a full-scale, high-fidelity simulation-based major injury scenario for assessment where their performance was scored (described below). For the two groups who were allocated to receive the practical training course, the course was completed 4–6 weeks before the simulation-based assessment.

With advertising for participants, and with cooperation of large building companies as well as local builder apprenticeship training programs in Sweden, individuals working on building sites as employees or apprentices were screened and recruited with cooperation of their employers. Not having Swedish language was an exclusion criterion.

For the planning and design of the scenario and simulation, with assessment and data collection, an expert group was formed consisting of anesthesiologist, emergency nurse, ambulance nurse, trauma surgeon and safety experts from the construction industry. The expert group designed a scenario that was realistic for the construction industry workplace environment. The expert group designed a 10-minute scenario with one injured person, 35 years old, previously healthy with multiple injuries caused by falls from 5 m. The scenario contained 2 critical diagnoses, catastrophic bleeding and occluded airway, where early (first minutes) recognition and treatment of these can be presumed to be potentially life-saving. The premise was that it was going to take a long time for ambulance personnel to arrive on site. The focus was on what the participants would do during the first 10 min. The simulation was conducted with the help of a facilitator who had also been the first response course instructor, and a high-fidelity wireless computer operated human patient simulator (HPS) which was preprogramed with pathophysiology parameters specific for this scenario (see Table 1). Two behaviors were included in the scenario as secondary elements: a safety check for the injury event place, and systematic head to toe survey and re-evaluation, commonly referred to as SCABCDE, was used to be able to detect other injuries.

**Table 1:** Scenario- critical diagnoses and assessment preprogrammed and set up in human patient simulator

Name	Critical diagnosis, assessment	Human patient simulator	Primary critically outcome
S -Safety	Safe injury- event site expected to be visually assessed 360 degrees		
C -Catastrophic bleeding	Catastrophic bleeding right femoral artery	0.5 L of ‘blood’ was out on the ground at the source of bleeding, as well as pulsating bleeding	Direct manual pressure over bleeding source < 60 s or Tourniquet < 90 s
A-B -Airway -Breathing	Blocked airway Apnea without airway management	Simulated chewing gum applied in the upper airway. Recorded sound with signs of blocked airway, released when airway was secured. Cyanosis when blocked airway, blue light in the face, removed when secured airway. Air stream on exhalation at secured airway The chest moves up and down at the open airway, Respiration rate 20/minutes	Inspect the oral cavity, Jaw Thrust < 90 s or Oro-pharyngeal airway < 90 s
C -Circulation	Hypotension and tachycardia	Heart rate 130/minutes. Blood pressure 70/40 mmHg Pale skin color and simulated sweat. Palpable pulse A, carotid	
D -Disability	Unconscious does not react to pain. Equal pupils on the right and left sides	Does not respond to contact. Does not react to painful stimuli. Pupils same size, responsive and react to light	
E Exposure	Wound injury in the back of head. Inwardly rotated legs. Blood on the legs.	Made-up wound injury back of the head. Legs inwardly rotated	Secondary outcome Examine the entire body head to toe, Pelvic sling in 10 min, mean time until completed

The expert group prepared a simulation facilitator`s manual with the aim of standardizing the simulation. The manual described the expected life-saving measures, which were required in order for the injury figure’s condition to improve in the simulation. The manual also described types of help the facilitator could supply to the study participants, for example in the case where expected life-saving measures were not addressed, so-called ”Lifesaver” [21] hints could be provided to allow the simulation to progress, even if the participants were unable to demonstrate one or the other critical behavior in the first phase of the scenario.

The video support content and possible interventions were based on a pilot project [13]. The 6-hour advanced first aid course, along with the medical equipment in the scenario, were developed in this same pilot project. The medical equipment kit was available to all the participants during the simulation-based assessment. This included checklists for field vital sign assessment and as well as instructions for video communication with remote ambulance personnel.

The 6-hour practical advanced first aid course included an emphasis on assessment of the injured party and advanced critical life-saving procedures (direct pressure or tourniquet, jaw thrust, oro - pharyngeal airway, laryngeal mask airway, bag-mask- ventilation, CPR and defibrillation and pelvic sling) with accompanying systematic checklist (SCABCDE). The training course also included introduction of the telemedicine supporting system. The course began and ended with a 10-minute trauma response exercise, event-based training, and participant reflection in groups. The procedures were first introduced through instruction film and instructor demonstration, then the students practiced on a patient simulator, with feedback from the instructor.

The medical kit included biosensors, with the capacity to measure and transmit the following medical parameters: breathing rate, oxyhemoglobin percent or saturation (SpO_2_), heart rate, blood pressure, electrocardiogram selected leads, and temperature. The participants had the option to demonstrate the situation using video in the telephone or connecting biosensors. Though not part of this study primary analysis, there was a pre-programmed and for-purpose designed smartphone function available, to connect to a server where medical instrument measurements in the simulation could be transmitted to the simulated ambulance personnel.

Based on direct observation and supported by the video recording of the participant performance during the standardized simulation, participant behaviors were scored using a pre-defined set of outcomes and time intervals. The scenario aimed to present clear signs of immediate life-threatening injuries in order to test for recognition and intervention behaviors, correct or incorrect. The whole scenario included the first 10 min of primary systematic prehospital trauma care, though the critical behavior period was defined as the first 90 s for 2 categories of primary responses. A detailed scoring protocol was developed which followed the expected measures in the standardized scenario. There were always 2 assessors for the primary and secondary outcomes, though exact times when these outcomes were achieved was confirmed by one assessor using the video recording. The outcomes were simple categories of responses, and agreement was required between assessors on whether or not the outcome was achieved. The assessors were not blinded since they could observe the interventions at the same time as the outcomes were assessed.

Before each group started the simulation-based assessment, the participants were given a standardized short orientation and introduction, including details about the training environment and how the simulator worked. After introduction, each participant completed a pre-assessment registration where they recorded their sex, age, years in the profession, and any resuscitation training they had prior to this study. The simulation was conducted in a standardized room where the human patient simulator was lying on their back on the floor. Next to the simulator was a medical kit with the above-described equipment, telephone, biosensors, and checklists/action cards. All groups had the same opportunity to use the equipment freely. An instructor conducted the assessment simulation scenario. An emergency nurse acted as (1) simulated emergency call center operator and (2) simulated ambulance nurse for remotely video support. Before the test started, all groups had been informed to simulate a call to the Swedish central emergency alarm phone number/112 on arrival at the scene of injury event. The two groups allocated to receive video support were directly ‘called’ (telephone) by a simulated ambulance nurse, immediately after the call to 112, and the video distance support was started using the checklist SCABCDE. The scenario/assessment period was stopped at 10 min. At this point, the group was asked to report back to the ambulance nurse on what they understood in the scenario, and what they had done as far as resuscitation. There were 2 primary outcomes, both within the first 90 s. These were early correct bleeding control by manual pressure within 60 s or applied tourniquet within 90 s, yes or no, and then correct identification of occluded airway with behavior to manage airway obstruction, also within 90 s. Secondary outcomes were assessed within the 10-minute time frame, and these included the following: correct top-to-toe examination finding wound injury in back of the head, also a categorical variable yes or no, time to completion of top-to-toe examination mean in seconds, correct fixation of pelvic injury a pelvic sling, categorical variables, yes or no, and time in seconds to fixating a pelvic fracture with a pelvic sling.

### Power calculation for a sample size

After intervention, either training course, ambulance tele-support, or both, the correct response for the critical behaviors was expected to be approximately 90% based on earlier course experience. The expected baseline rate for responses or behaviors from completely unschooled or untutored participants was expected to be not more than 25%. This meant an estimated or anticipated difference in frequencies correct versus incorrect responses or proportions of 0.65, with power to detect a true difference of 80% and a 2-sided ‘alpha’ of 5% (0.05). This calculation indicates that a minimum of 8 sets of participants or groups should be in each paired analysis. A sample size of 10 for each set of groups was chosen, to allow for dropout.

### Data management

A total of 40 simulation assessments were observed and recorded with two cameras and from two angles. Exposures were pseudoanonymized for the assessments, and for the analysis of the videos. The videos and observation protocol were encoded and stored on a server with a coded password to which only primary investigator had access.

### Statistical analysis

Analysis was conducted using IBM SPSS statistics, version 28. Descriptive statistics are presented both at individual level and at group level. Group comparisons for the primary outcome frequencies correct and incorrect were done using the Fisher’s exact test, based on the small sample size. For continuous variables where an approximately normal distribution could be assumed, an independent samples standard t-test was used. Maximum time in the assessment scenario was 10 min or 600 s, and groups that did not complete the expected procedure were assigned 600 s for that outcome. Significant differences between groups were identified when the p value was less than 0.05.

### Ethical consideration

The study was approved by the Swedish Ethical Review Authority (document number 2021-05774-01). All participants provided informed consent before entering the study.

## Results

### Study participation-description

Data was collected in Northern Sweden between February and June 2022. A total of 90 individual participants completed the study, all in groups of 3. One participant in Group 1, and two participants in Group 2, did not answer the background questions of the survey. Demographics are shown in Table 2.

**Table 2:** Participant background data

Measures	Group 1/4 (n = 10) Individuals (n = 29)	Group 2 (n = 10) Individuals (n = 28)	Group 3 (n = 10) Individuals (n = 30)
Female/Male	2/27	7/21	7/23
Age (mean ± sd)	28 ± 14	40 ± 13	28 ± 14
*Previous Training			
First aid trauma course	2	2	1
CPR training course	4	5	1
CPR + defibrillation course	1	1	1

*Previous training was defined as completed training course, First aid trauma course (ABC, ABCDE, SABCDE) within 2 years, Cardiopulmonary training course with or without defibrillator (CPR, CPR + Defibrillation within 2 years). The youngest person in the study were 18-year-old and the oldest 60 year

### Primary outcomes

For the first primary outcome, first without any preparation or training, there were almost no correct behaviors observed for the baseline assessment, for both bleeding control and airway management. Then, concerning interventions, video support by itself with no course preparation (Group 2) was associated with better critical bleeding control compared to those who had neither course or video support (Group 1) (9/10 vs. 1/10, p = 0.001) (Table 3). For bleeding control, the pre-treatment course and video support combined Group 3 was not statistically different in performance compared to those that had the course by itself (Group 4) or video support by itself (Group 2) (8/10 vs. 5/10, p = 0.35, and 8/10 vs. 9/10, p = 1, respectively), and all groups had a high proportion of correct behaviors.

**Table 3:** Primary critical outcomes (bleeding or airway control < 90’’)

	Group 1	Group 4	Group 2	Group 3	*P value*
	Baseline	Training course only	Video support only	Video support + training course	
Bleeding control < 90’’	1/10	5/10			*0.14*
	1/10		9/10		*0.001*
		5/10	9/10		*0.14*
		5/10		8/10	*0.35*
			9/10	8/10	*1*
Airway control < 90’’	0/10	9/10			*< 0.001*
	0/10		4/10		*0.02*
		9/10	4/10		*0.06*
		9/10		8/10	*1*
			4/10	8/10	*0.17*

Group 1 received no telemedicine support and with no course training. This same group then later (after the assessment) participated in the training course and had a second assessment 4 to 6 weeks after their course, and this assessment group was called Group 4.

Concerning airway management responses, only 40% of participants in Group 2 (video support only) correctly managed the airway critical step, despite video support. The course by itself (Group 4) was superior to no course (Group 1) where neither had video support for airway management (9/10 vs. 0/10, p < 0.001), but not statistically different from video support by itself (9/10 vs. 4/10 respectively, p = 0.06).

### Secondary outcomes

Use of the vital sign biosensors in the scenario by the study participants was zero in groups 1, 2 and 4. Group 3 connected biosensors to the ‘injured’ in 10/10 groups, but only very late in the 10-minute scenario, and there was no attention for any group concerning biosensor readings. Concerning the head-to-toe examination, in the baseline assessment there was no group that performed this (Table 4). Further, video support with or without a pre-treatment course (groups 3 and 2) was associated with better performance compared to the those with no video support (Group 4) (9/10 vs. 2/10, p = 0.01; 8/10 vs. 2/10, p = 0.02, respectively). Time to the outcome event was in line with the frequency comparisons between groups. Concerning the behavior establishing a pelvic sling to limit suspected internal bleeding, the video support plus course group had perfect performance (10/10 groups) while both the course by itself and the video support by itself groups had half or more showing this behavior.

**Table 4:** Secondary outcomes

	Group 1	Group 4	Group 2	Group 3	*P value*
	Baseline	Training course only	Video support only	Video support + training course	
Head-to-toe examination (number correct) and Time (mean, sd, seconds)	0/10	2/10			*0.47*
		541 ± 126			
	0/10		8/10		*0.001*
			410 ± 109		
		2/10	8/10		*0.02*
		541 ± 126	410 ± 109		*0.02*
		2/10		9/10	*0.01*
		541 ± 126		288 ± 115	*< 0.001*
			8/10	9/10	*1*
			410 ± 109	288 ± 115	*0.03*
Pelvic sling (correct)	0/10	6/10			*0.01*
		450 ± 144 s			
	0/10		5/10		*0.03*
			579 ± 25		
		6/10	5/10		*1*
		450 ± 144	579 ± 25		*0.01*
		6/10		10/10	*0.09*
		450 ± 144		358 ± 54	*< 0.001*
			5/10	10/10	*0.03*
			579 ± 25	358 ± 54	*< 0.001*

## Discussion

The main findings were that participants, untrained or unprepared laypersons active in the construction industry, showed a low ability to manage catastrophic bleeding and occluded airway in a 10-minute simulated accident scenario. Participants had a low degree of current practical training in first aid trauma care and lifesaving before entering the study, so the study’s possibility to assess effects of training and telemedicine support were good. Preparation or support through either the 6-hour practical training course in life-saving procedures, or medical telemedicine support from distance through realtime video support, or a combination of both, was associated with increased effect in carrying out life-saving procedures while waiting for an ambulance to arrive at the scene of a simulated injury workplace event. These findings are in line with those from Bakke et al. 2013 who found that only 35% of the laymen had a practical training in first aid competences, and that laymen who had documented practical training manage first aid for injuries more effectively than those who did not have documented education [12].

In this simulation-based assessment for a serious workplace injury event, we could see significant improvements in managing a catastrophic bleeding and occluded airway, after practical training or with video support from ambulance personnel. Responses to manage catastrophic bleeding with direct pressure or with a torniquet within 90 s were best. Results for managing an occluded airway with video support were better compared to no course or support at all, but not clearly better than for those with the training course.

The effect of a training course/education is expected to decrease over time without recurrent training or repetition [22–23]. For companies in the construction industry, it can be a challenge to dedicate time for regular refresher practical training in advanced first aid procedures. External real-time telemedicine resources to support local layperson responses to serious injury events could be a practical way to improve early response effectiveness even for those who have not had recent first aid courses. The findings here show that support through video calls provides meaningful benefit to layperson performance, independent of preparatory practical training or not. Practical training combined with video support may provide additional benefit, though this study design was not optimal to assess this.

Several studies have validated the concept of medical support from distance, through a communication-distance solution, telephone, or video system, for instance in connection with CPR, trauma management, or assessment of various medical conditions such as stroke [14–17, 24, 25]. Nord-Ljungquist et al., 2020 studied dispatcher support to layperson by phone, for CPR before an ambulance arrived [25]. Those findings showed difficulty in getting layperson to correctly manage an airway blockage, which our results confirm. Landgraf et al., 2019 reported on a telemedical support system with offshore emergency scenarios and quality of medical first response by medical non-professional comparing to medical professionals, and found that the supported group required more time to act compared to non-supported [24].

In our scenarios/simulation sessions where participants were supported by video, the success rate for managing an occluded airway with the jaw-thrust procedure was not as high as expected. This possibly could be due to the complexity or unfamiliarity with evaluating and managing an occluded airway. We also observed that where the groups had practical course experience, sometimes they focused on the experience they seemed to remember related to the training course, which may have hindered video communication to guide intervention. An interaction between these different exposures could have led to dilution of possible benefit from the combined interventions. These observations are also in line with results from Linderoth et al., 2021 [14], where they concluded that in order to support the layperson by video, dispatchers at the emergency call center-112 must understand the situation in order to best facilitate the layperson in their actions. It appears that video support can change the emergency response, though it is challenging to use this approach to advantage within the context of existing dispatch protocols [26].

The interaction between dispatcher and layperson is important, but, in addition, interactions between the laypersons on-scene are also important. Teamwork within the groups was observed when there was a video dialogue with ambulance personnel. One layperson needed to focus on the smartphone and film the injured person, while at the same time listen and try to understand the advice from the ambulance personnel, and then communicate this to the laypersons in the team. Specific team non-technical performance, including communication, situational awareness, and distributing workload in the team, was not assessed in this study. Non-technical performance for both layperson and ambulance personnel could be relevant for future testing of a video support system for this type of response.

None of the groups spontaneously connected biosensors (heart rate, pulse oximetry, blood pressure measuring devices) which could transmit signals to the video supporter. Use of these was taught in the course. Even with video support, implementation of vital sign measurement did not come up until bleeding, airway, and even head-to-toe assessment and pelvic sling steps were completed. This meant that measuring and monitoring vital signs (and transmitting) in practice for this scenario came later, if it was done. These biosensor signals were still appropriate for more informed video support, and not only for the ensuing phase. As assessed here, vital signs measurement, or impact on measuring vital signs on the course of video support, could not be assessed. Vital sign assessment should be a priority early in this type of scenario. This could be something that can be emphasized in both training courses and video support tactics.

The study context was based on Swedish construction industry conditions. There are other initiatives that have focused on the effects of collaboration between layperson and professional rescue personnel while waiting for an ambulance or fire brigade. One initiative is the Civil Response Person and In Wait for Ambulance [27–28]. The concept is that individuals with established technical means to receive an ‘alarm’ can be sent to a nearby accident site as prepared layperson responders before ambulance or fire brigade personnel arrive. Both this alert concept as well as direct two-way interaction have been tested in the community to facilitate layperson responses for early management of critical situations such as cardiac arrest or traffic accidents. Some reported experience is that there is sometimes insecurity among laypersons in these actions when acting by themselves, though not after first contact with ambulance personnel [27, 29]. Further research in this area could focus on evaluation of a supporting model including dispatchers, ambulance personnel, and interaction with laypersons, to optimize video support for lifesaving procedures.

### Limitation

In this study, participants were inexperienced and untrained in this specific context, including in working or assessment using a full-scale high-fidelity simulator. Working in groups, there appeared to be good immersion into the clinical scenarios, with no difficulty with ‘suspension of disbelief’ concerning the simulation. The video connection to ambulance personnel used here was a nono-commercial prototype, though commercial products for this purpose are expected to be widely available soon. The study groups were small, meaning that there can be imprecision in estimating effect sizes of the interventions. The preparatory course and the ambulance personnel protocolized communication can be updated and improved prior to future studies of efficacy and implementation. Since the only group that connected the biosensors to the ‘injured’ was the one that had both the training course and video support with ambulance personnel, there were limitations in assessing how the biosensor-based information might influence behavior. This finding though could inform future study design where biosensor information is central to the study question. The choice to assess learning and behavior related to the interventions using simulated injury events, rather than actual events, is a first step in studying these interventions, given that real-world serious trauma events at building sites are not common and not planned. Still, if and when these types of interventions might be implemented by builder organizations, the practical results will need to be assessed as part of implementation studies.

## Conclusion

These findings show that for laypersons (here construction industry employees) and first responsers in a serious injury scenario during the wait for ambulance arrival, airway management and active bleeding control, are improved by live video support, including if these actions have been trained beforehand.

### Electronic supplementary material

Below is the link to the electronic supplementary material.

Supplementary Material 1

## Data Availability

The simulation and assessment protocol is available as a supplemental file. The dataset is available from the corresponding author on reasonable request.
