# Supplementary material for: Effects of an advanced first aid course or real-time video communication with ambulance personnel on layperson first response for building-site severe injury events: a simulation study
Source: BMC Emerg Med. 2024 Jan 7;24:2. doi: 10.1186/s12873-023-00917-4 (PMC10773037; doi:10.1186/s12873-023-00917-4)
Supplement: Supplementary file 1 — Supplementary Material 1 [file 12873_2023_917_MOESM1_ESM.doc]

**TRIAL PROTOCOL**

| **Observations, Variable list** | **Time in seconds** | **Score** |
| --- | --- | --- |
| *Arrive at the scene of the accident – ambulance call up* |  |  |
| Checks that the location is secure **S 360 degrees around the accident scene** |  | 1: Yes  0: No |
| Assessment C  *Noted critical bleeding* |  | 1: Yes  0: No |
| □ Direct manual pressure over bleeding source |  | 1: Yes **60 sek or <**  0: No |
| □ Pressure bandages |  | 0: No  1: Yes |
| □ Torniquet |  | 1: Yes **90 sec or <**  0: No |
| □ Protect the cervical spine |  | 1: Yes  0: No |
| Assessment of **A-Airway** |  |  |
| □ Talk to the patient |  | 1: Yes  0: No |
| □ Inspect the oral cavity |  | 1: Yes  0: No |
| □ Listen for breathing sounds |  | 1: Yes  0: No |
| □ Feel after exhalation |  | 1: Yes  0: No |
| □ Chin lift (CPR method) |  | 1: Yes  0: No |
| □ Jaw Thrust |  | 1: Yes **90 sec or <**  0: No |
| □ Oro-pharyngeal airway |  | 1: Yes **90 sec or <**  0: No |
| □ Laryngeal mask airway |  | 1: Yes  0: No |
| Assessment of **B-Breathing** |  |  |
| □ Inspection chest movement |  | 1: Yes  0: No |
| □ Feels the movement of the chest, symmetry |  | 1: Yes  0: No |
| □ Assesses respiratory rate/ minute |  | 1: Yes  0: No |
| Fixed **B**  **Secure free airway** |  | 1: Yes  0: No |
| Assessment of **C-Circulation** |  |  |
| □ Reassessment of bleeding |  | 1: Yes  0: No |
| □ Pulse control Arteria radialis |  | 1: Yes  0: No |
| □ Pulse control Arteria carotid |  | 1: Yes  0: No |
| □ Capillary refilling |  | 1: Yes  0: No |
| □ Skin temperature |  | 1: Yes  0: No |
| □ Skin color |  | 1: Yes  0: No |
| Assessment of **D-Disabillity** |  |  |
| Assessment of Consciousness □ ACVPU |  | 1: Yes  0: No |
| Assessment pupils □ PEARL |  | 1: Yes  0: No |
| **E-Exposure** |  |  |
| □ Examine the entire body head to toe |  | 1: Yes  0: No |
| □ Protect against cooling |  | 1: Yes  0: No |
| □ Pelvic sling |  | 1: Yes  0: No |
| □ Connect biosensor for monitoring |  | 1: Yes  0: No |
| REEVALUATION C-ABCDE |  | 1: Yes  0: No |

□ Primary critically outcome in this study

□ Secondary measure in this study

□ Not included in this study
